# First-ever ASM Global Research Symposium on Microbes in Human Health in Tsinghua, Beijing, China

**DOI:** 10.1128/msphere.01092-24

**Published:** 2025-02-24

**Authors:** Sai Luo, Ya-Ting Wang

**Affiliations:** 1 Center for Infection Biology, School of Basic Medical Sciences, Tsinghua University, Beijing, China; 2 SXMU-Tsinghua Collaborative Innovation Center for Frontier Medicine, Shanxi Medical University 74648 , Taiyuan, Shanxi Province, China; University of Michigan, Ann Arbor, Michigan, USA

**Keywords:** innovations in clinical microbiology, bacterial and viral pathogenesis, pathogen–host dynamics, vaccines and therapeutics

## Abstract

The American Society for Microbiology Global Research Symposium on Microbes in Human Health, hosted in partnership with Tsinghua University, was held in Beijing, China, on 25 to 27 September 2024. The conference provided an international forum for microbiologists from different disciplines to present and discuss new findings. The meeting covered a wide range of topics, spanning across bacteria, virus, fungi, and hosts. This report summarizes the presentations and emerging themes.

## INTRODUCTION

The meeting was chaired by Jing-Ren Zhang and co-chaired by Qiang Ding, Tsinghua University, China, and covered an exciting range of topics across the scope of research on microbiology and human health. It comprised three keynote lectures and 34 talks in four thematically organized sessions of “Innovations in Clinical Microbiology,” “Bacterial and Viral Pathogenesis,” “Pathogen-Host Dynamics,” and “Vaccines and Therapeutics.” The meeting also included an extensive poster session. In this review, we attempt to convey the depth and breadth of the topics that were presented during this meeting and provide a brief summary of recent developments and emerging trends in the field.

A central theme of the meeting emphasizes the importance of scientists working together to advance science without barriers to collaboration. The COVID-19 pandemic underscored a crucial lesson: infectious diseases know no borders. Just as microbes recognize no boundaries, science and scientists must transcend them, fostering unrestricted cooperation to address global challenges effectively.

To foster strong relationships and collaboration in China, ASM has chosen to partner with Tsinghua University, one of the world’s most prestigious and influential institutions. Established in 1911, Tsinghua University is renowned for its academic excellence, innovative research, and dedication to cultivating future leaders. Consistently ranked among the top global universities, Tsinghua University excels in science, engineering, and technology.

The host of the meeting, the School of Basic Medical Sciences (BMS) at Tsinghua University, is a leading institution for medical education and research in China. BMS is committed to training exceptional professionals in basic medical sciences, including infectious diseases, immunology, cancer biology, neurodegenerative diseases, and molecular biology. The school conducts cutting-edge biomedical research, advancing global health through interdisciplinary collaboration and state-of-the-art facilities. With its contributions to medical science and healthcare, BMS continues to play a pivotal role in addressing global health challenges.

In this report, we will provide a recap of ASM talks in three major areas: bacterial, fungi, and virus. The bacterial talks predominantly focused on pressing topics of antimicrobial resistance, bacterial pathogenesis and vaccines, and host innate immune defense against bacterial pathogens. The central themes on discussions on fungi revolved around fungi drug resistance. The topics of virology talks included viral pathogenesis, host–virus dynamics, viral vaccine and antiviral therapeutics, and epidemiology and surveillance of viruses.

## BACTERIAL ANTIMICROBIAL RESISTANCE AND CLINICAL MICROBIOLOGY

Antimicrobial resistance (AMR) is a growing threat to global public health, linked to approximately 4.95 million deaths in 2019, according to a World Health Organization (WHO) report (1). A comprehensive study in 2024 examined the global burden of AMR from 1990 to 2021, projecting that by 2050, AMR could result in over 10 million deaths annually (2). This alarming estimate solidifies AMR as one of the most critical health challenges of the 21st century. Among pathogens, methicillin-resistant *Staphylococcus aureus* (MRSA) showed the greatest increase in attributable burden across all age groups. In 2021, the pathogens causing the highest number of deaths attributable to AMR were *Klebsiella pneumoniae*, *Staphylococcus aureus*, *Escherichia coli*, and *Mycobacterium tuberculosis*. In 2024, the WHO updated its bacterial priority pathogen list to guide research and strategies for AMR prevention and control. Gram-negative bacteria resistant to last-resort antibiotics—such as *Acinetobacter baumannii* and various species within the *Enterobacterales* order—along with rifampicin-resistant *M. tuberculosis*, were categorized as critical priorities. These pathogens pose significant risks due to their capacity to transfer resistance genes, the severity of the diseases they cause, and their substantial global burden, particularly in low- and middle-income countries (LMICs) (1). Addressing these critical challenges will require concerted global efforts to develop effective diagnostic, therapeutic, and preventive measures.

AMR is a multifaceted and diverse phenomenon influenced by a range of biological, social, and environmental factors. Dr. Cesar Arias, from Houston Methodist Hospital, USA, and editor in chief of *Antimicrobial Agents and Chemotherapy*, highlighted the regional differences in the genomic epidemiology of MRSA in Latin America. For instance, clonal cluster 5 (CC5) MRSA is predominant in bloodstream infections across the region, except in Colombia and Ecuador, where the USA300-LV lineage is now dominant (3). This clonal replacement underscores the dynamic nature of MRSA epidemiology, emphasizing the necessity of continuous molecular surveillance to monitor and respond to evolving trends. Advanced phylogenomic analyses, such as studies of the arginine catabolic mobile element (ACME) and the novel copper and mercury resistance element (COMER), have provided insights into the evolution of community-associated MRSA (CA-MRSA). Comparative genomic studies between the USA300 clone responsible for the CA-MRSA epidemic in the United States and MRSA isolates from South America revealed the existence of two parallel USA300 epidemics in North and South America, both originating from a recent common ancestor. These findings illustrate the complexity of molecular epidemiology and its potential implications for clinical outcomes. Carbapenem-resistant *Klebsiella pneumoniae* (CRKP), recognized by the WHO as a critical AMR threat, further highlights the regional variability in AMR dynamics. CRKP epidemics demonstrate significant differences across regions in patient baseline characteristics, clinical outcomes, and bacterial features (4). These variations suggest that insights gained from one region may not always be applicable to others. This growing body of evidence reinforces the importance of integrating genomic surveillance, regional epidemiological studies, and clinical research to combat AMR and improve patient outcomes worldwide.

Advancing drug resistance omics studies is pivotal to unraveling the sources of resistance genes and transmission mechanisms, which can aid in developing rapid diagnostic techniques for antimicrobial-resistant bacteria. Carbapenem-resistant Enterobacterales (CRE) often harbor carbapenemases—enzymes belonging to molecular classes A, B, and D β-lactamases. Class A and D enzymes utilize a serine-based hydrolytic mechanism, whereas class B enzymes, known as metallo-β-lactamases, rely on zinc in their active sites. Among class A carbapenemases, *Klebsiella pneumoniae* carbapenemase (KPC) is a prominent driver of carbapenem resistance in Enterobacterales (5). Dr. Hui Wang from Peking University People’s Hospital, China, presented recent efforts to elucidate the transmission dynamics of KPC by mining global epidemiological data of associated plasmids. This work uncovered intra-*K. pneumoniae* and cross-genus KPC transmission patterns, advancing our understanding of its spread (6). Additionally, Dr. Wang highlighted the CRE surveillance network in China, established in 2011, which spans over 90 hospitals across 28 provinces. This network has provided critical insights into regional CRE epidemiology over the past decade. From 2011 to 2021, *K. pneumoniae* emerged as the predominant CRE species in China, with KPC-2 being the primary resistance mechanism (7). Among the 277 sequence types (STs) of CRKP, ST11 has remained the dominant clone. Originating in the United States, ST11 expanded in China during the mid-2000s but has since been supplanted by the sub-lineage ST11-KL64. This sub-lineage is rapidly expanding in China and exhibits higher mortality rates, particularly in elderly patients, due to its enhanced resistance to host immune defenses such as phagocytosis. Phylogenetic analyses revealed that ST11-KL64 CRKP has acquired a pK2044-like virulence plasmid from ST23-KL1 hypervirulent *K. pneumoniae*. Interestingly, this plasmid features a consistent 2,698-bp deletion that regulates methionine metabolism to enhance the antioxidant capacity of the strain. This adaptation improves the survival of hypervirulent CRKP within macrophages, contributing to its heightened pathogenicity (8). These findings underscore the critical role of genomic surveillance in understanding the evolution and dissemination of AMR pathogens, providing a foundation for developing targeted strategies to combat this global health threat.

Dr. Alessandra Carattoli from Sapienza Università di Roma, Italy, highlighted the critical role of plasmids as cross-ecological vectors of AMR, bridging humans, animals, and the environment. The success of plasmids in spreading resistance is influenced by factors such as their host range, conjugation rate, and the fitness cost imposed on the bacterial host. Carattoli’s discussion centered on CPE, particularly those producing metallo-β-lactamases (MBLs), which represent some of the most challenging antibiotic-resistant pathogens. These bacteria are responsible for outbreaks of difficult-to-treat nosocomial infections worldwide. Plasmids have been identified as key players in the acquisition and dissemination of carbapenemase genes, such as *bla_OXA-48_* and *bla*_NDM_. In *K. pneumoniae*, a wide range of plasmid families encoding AMR genes has been documented. Of particular note, an outbreak of New Delhi metallo-β-lactamase-positive CPE (NDM-CPE) occurred in Tuscany, Italy, in 2018. This outbreak was caused by *K. pneumoniae* sequence type 147 (ST147) producing NDM-1. Analysis revealed the presence of FIB(pQil)-type multiresistance plasmids carrying *bla*_NDM-1_. Additionally, a transferable chimeric plasmid, combining virulence elements from hypervirulent *K. pneumoniae*, was identified. This chimeric plasmid encoded multiple resistance and virulence determinants, representing a significant threat due to its ability to propagate both pathogenicity and drug resistance traits (9). Carattoli’s findings underscore the importance of understanding plasmid dynamics in AMR dissemination. By elucidating how plasmids facilitate the spread of resistance genes across ecosystems and bacterial populations, her work provides valuable insights into combating the global AMR crisis.

In response to the growing threat of AMR, there is an urgent need to explore alternative strategies for eliminating bacterial pathogens. Dr. Rubing Liang from Shanghai Jiaotong University, China, presented an innovative approach involving tellurite-mediated bactericidal effects. Tellurite, a metalloid with antimicrobial properties, exerts its bactericidal effect by inducing intracellular acidification. This acidification activates the bacterial acid tolerance pathway, which disrupts cellular homeostasis in multiple ways. Key outcomes include magnesium depletion and the subsequent blockade of protein synthesis, effectively halting bacterial growth and survival. Leveraging tellurite as a novel antimicrobial agent provides an alternative means to target resistant bacterial strains.

## BACTERIAL PATHOGENESIS AND VACCINES

Dr. Qijing Zhang from Iowa State University, USA, delivered a keynote address on the pathogenesis of *Campylobacter jejuni*-induced systemic infections, highlighting its emergence as a major foodborne human pathogen with broad host specificity and increasing resistance to fluoroquinolone (FQ) antimicrobials. *Campylobacter jejuni* is a Gram-negative, spiral-shaped, microaerophilic enteric pathogen. Infections are typically acquired through contaminated food, water, or beverages. Zhang’s presentation focused on a hypervirulent, tetracycline-resistant zoonotic *C. jejuni* clone, sequence type 8 (ST-8), which has been identified as the predominant cause of ruminant abortions in the United States and implicated in foodborne disease outbreaks in humans (10). ST-8 *C. jejuni* has a unique ability to translocate across the intestinal tract, resulting in systemic infections and fetoplacental infections in pregnant animals. Using mouse and guinea pig models, they identified the bacterial capsule as indispensable for systemic infection and bacteremia (11). However, despite its importance, capsule sequences remain highly conserved across virulent and nonvirulent clones, indicating that capsule alone does not account for hypervirulence. A novel “directed genome evolution” strategy revealed that single-nucleotide polymorphisms (SNPs) in the *porA* gene, encoding the major outer membrane protein, are responsible for ST-8’s enhanced virulence (12). RNA-seq analysis of infected aborted placental tissues demonstrated upregulated inflammatory cytokine production, further underscoring the role of *porA* mutations in pathogenesis. Whole-genome SNP analyses suggest that the clonal expansion of ST-8 is a relatively recent event. The acquisition of *porA* mutations and the integration of tetracycline-resistance genes indicate that tetracycline usage has likely driven the emergence and spread of this hypervirulent clone (12, 13). The rapid spread and pathogenicity of ST-8 highlight the need for updated vaccines. Commercial vaccines have proven ineffective against ST-8-induced abortions in sheep (14). However, experimental homologous bacterins have shown significant promise, providing protection in pregnant ewes against *C. jejuni*-induced abortion (14). Zhang’s findings emphasize the urgent need for continued research into the molecular mechanisms driving *C. jejuni* hypervirulence and resistance. Developing targeted vaccines and refining antimicrobial strategies are critical for mitigating the impact of hypervirulent *C. jejuni* clones on both animal health and human foodborne illness.

The WHO recently highlighted the potential of vaccines to combat AMR, estimating that widespread vaccine use could reduce global antibiotic consumption by 22% annually (15). Dr. Jing-Ren Zhang from Tsinghua University, China, presented recent findings on the immune mechanisms underlying the differential efficacies of pneumococcal polysaccharide vaccines (PPSV23) and polysaccharide–protein conjugate vaccines (PCV13) *Streptococcus pneumoniae* vaccines. Contrary to the long-standing paradigm that migrating phagocytes mediate immunity against bloodstream pathogens, Zhang’s research reveals that immunity elicited by these vaccines primarily operates in the liver. The higher efficacy of PCV13 is linked to enhanced bacterial capture by hepatic endothelial cells, a process facilitated by IgG–FcγRIIB interactions (16). In contrast, PPSV23 relies solely on Kupffer cell activation through IgM-induced complement pathways, without endothelial cell involvement. These findings provide crucial insights into how vaccine-induced immunity functions and underscore the superior protective mechanisms of PCV13 against bloodstream *S. pneumoniae* infections.

Despite PCV13’s greater efficacy, its high cost limits accessibility, especially in low-resource settings. Dr. Jinbo Gou from CanSino Biologics Inc., China, shared promising advances in protein-based pneumococcal vaccines (PBPVs). In Phase Ib trials involving adults over 50 years of age, PBPVs demonstrated a better safety profile and elicited higher antibody levels against protein antigens compared to PPSV23 (17). These developments suggest that PBPVs could offer a more affordable and effective alternative to current vaccines, addressing limitations in accessibility and efficacy. The findings presented highlight the importance of understanding protective immune mechanisms to inform the design of next-generation vaccines. Improved vaccines could enhance global efforts to combat AMR by reducing the reliance on antibiotics and providing broader protection against pneumococcal infections.

## HOST INNATE IMMUNE DEFENSE AGAINST BACTERIAL PATHOGENS

While vaccines aim to enhance adaptive immunity to combat AMR, strengthening host innate immune defenses may also offer innovative antimicrobial strategies to mitigate AMR threats. Dr. Feng Shao from the National Institute of Biological Sciences, Beijing, China, presented compelling research on gasdermin activation and pyroptosis as critical components of host antimicrobial defenses and sepsis management. Gasdermin D (GSDMD) is a pore-forming protein that plays a pivotal role in pyroptosis, an inflammatory form of cell death (18, 19). Shao highlighted how GSDMD activation by the cytosolic lipopolysaccharide (LPS) sensor caspase-11 (Casp11) mediates blood–brain barrier (BBB) breakdown in response to circulating LPS or during LPS-induced sepsis. Unlike toll-like receptor (TLR)−4-induced cytokine responses, this pathway is specifically triggered by cytosolic LPS. LPS acts directly on brain endothelial cells, priming the expression of Casp11 and CD14, which in turn activates GSDMD (20). This leads to plasma membrane permeabilization and pyroptosis, contributing to BBB impairment during invasive bacterial infections. Experimental models *in vitro* and in mice demonstrated that GSDMD-mediated pyroptosis is a key driver of these pathological changes. These findings offer valuable insights into the molecular mechanisms underlying BBB disruption during sepsis and invasive bacterial infections. Targeting the Casp11–GSDMD axis may provide new therapeutic approaches for diseases of the central nervous system linked to BBB dysfunction, offering hope for conditions where current treatments are inadequate. By exploring innate immune defense mechanisms of pyroptosis, this research underscores the potential for developing novel antimicrobial strategies that complement existing adaptive immunity-based interventions.

Along the line of innate immune defense against bacteria, Dr. Yating Wang from Tsinghua University, China, discussed her recent research on inducing innate immune priming prior to infection to confer resistance to respiratory pathogens. In normal animals, innate immune priming was suppressed by autophagy in alveolar macrophages, rendering animals susceptible to infection. Mice lacking autophagy in myeloid cells demonstrated swifter and more efficient innate immune clearance of pulmonary MRSA infection, while minimizing lung damage and inflammation. Enhancing the primed state of innate immunity by blocking autophagy also provided protection against influenza A virus.

Dr. Haoran An from Peking University Health Science Center, China, focused on the role of *S. pneumoniae* capsules in enhancing bacterial survival during blood-borne infections (21). An’s study revealed that liver-resident macrophages, specifically Kupffer cells, capture less virulent capsule types of *S. pneumoniae*, but do not engage with those expressing more virulent capsule types. Contrary to prior studies highlighting the importance of neutrophils in eliminating blood-borne pathogens, An found that neutrophil depletion via antibodies did not impair the clearance of *S. pneumoniae* by Kupffer cells. In contrast, splenic red pulp macrophages were responsible for eliminating liver-resistant *S. pneumoniae* in the bloodstream. Interestingly, splenic clearance of more virulent capsule types of *S. pneumoniae* was dependent on natural antibodies binding to the pneumococcal cell wall phosphocholine, independent of neutrophils.

Dr. Peng Gao from the University of Hong Kong, China, introduced his research on how flufenamic acid enhances phagocyte intracellular killing of *S. aureus* by inhibiting the AgrA quorum sensing system. Gao’s study found that flufenamic acid works, in part, by activating the host’s NLRP3 inflammasome, a critical component of the innate immune system. This finding suggests that flufenamic acid could serve as a potential therapeutic agent for improving host defense against *S. aureus* infections by enhancing the ability of phagocytes to kill bacteria intracellularly.

## BACTERIAL COMMUNITY AND BACTERIOPHAGES

The gastrointestinal tract contains a complex ecosystem with abundant bacterial interactions. Dr. Jay Zhu from University of Pennsylvania, USA, discussed how dysbiosis, or imbalance in the microbiome, facilitates the colonization of *Vibrio cholerae* in the gastrointestinal tract. Dysbiosis and spent medium were shown to promote *V. cholerae* adherence to epithelial cells and enhance its motility. Zhu’s team identified *Streptococcus* as a predictive biomarker of *V. cholerae* infection in a cohort of household contacts of cholera patients (22). Further studies revealed that *Streptococcus* stimulates *V. cholerae* motility and adherence through the production of a short hydrophobic peptide (SVIP). *V. cholerae* motility is driven by its polar flagellum, which is powered by a sodium-conducting motor. SVIP increases the sodium level by docking onto the stators of this motor, thereby inducing *V. cholerae* motility. In the cohort of household contacts, the presence of SVIP in the microbiota predisposed individuals to cholera infections. This finding demonstrates how the microbial community composition, such as the presence of specific bacteria like *Streptococcus*, can influence the susceptibility to infections like cholera.

The oral microbiome is another complex microbial community that is temporally and spatially structured. But the overgrowth of oral bacteria can cause infection such as periodontitis and oral cavity. *Treponema denticola* (Td) is a “keystone pathogen” of periodontitis, which means a small population of this species supports and stabilizes a disproportionately large microbiota associated with disease states (23). Td is able to breach host immune defenses and becomes predominant in the periodontal pocket when dysbiosis and inflammation worsen. Dr. Chunhao Li from Virginia Commonwealth University, USA, analyzed secreted virulence factors in order to understand the mechanisms that allow Td to evade the host immune response. Li’s team identified TDE0362, a dual-domain virulence factor, as essential for Td pathogenesis. TDE0362 has two functions: first, it protects Td from serum killing by cleaving complement factors, and second, it inhibits neutrophil chemotaxis, which protects Td from neutrophil-mediated killing. This enables Td to survive and thrive in the periodontal pocket, even in the presence of an immune response, leading to persistent infection.

Biofilm community is a major form of existence for many bacterial pathogens during persistent infections (24). Dr. Jintao Liu’s group at Tsinghua University, China, investigated how biofilms interact with bacteriophages. They found that *K. pneumoniae* biofilms rapidly developed resistance to phages, not through the accumulation of mutations but due to the release of unassembled phage tail fibers from phage-lysed bacteria (25). These tail fibers degraded the bacterial capsule, which is essential for phages to bind and infect the bacteria, thereby protecting the bacteria from phage infection. The dense packing of bacteria in biofilms limits the diffusion of macromolecules into the biofilm. The study also demonstrated that phages could potentiate the effect of cellulase to destroy biofilms, which opens up possibilities for combining phages with a broad range of therapeutic macromolecules to enhance biofilm eradication. In another related talk, Dr. Jiafeng Liu from Tsinghua University also explored bacterial tolerance to phages. Unlike bacterial tolerance to antibiotics, which often involves increasing the lag time before bacterial growth, phage tolerance did not follow the same strategy. Instead, heat induced uniform phage tolerance in bacteria, suggesting that bacterial resistance to phages operates through mechanisms distinct from antibiotic resistance. Additionally, Dr. Shuai Le, from Key Laboratory of Microbial Engineering and Army Medical University, Chongqing, China, discussed the long-term coexistence of phages and bacteria in the human microbiome. His research focused on *Saccharibacteria* isolate TM7x, an ultrasmall bacterium, which protects its host bacterium, *Schaalia odontolytica* XH001, from lytic phage LC001 predation (26). The protective mechanism involved TM7x downregulating the host’s cell wall biogenesis of cell wall polysaccharides, which affects phage binding and prevents phage infection.

Ziyan Wang from Dr. Jintao Liu’s group at Tsinghua University studied nutrient gradients within biofilms, specifically focusing on phosphorus. Phosphorus is an essential nutrient for bacteria, yet biofilms continued to grow even after phosphorus removal. Wang’s research found that biofilm expansion was supported by reactive oxygen species (ROS)-mediated cell death within the biofilm. Phosphorus starvation induced cell death in bacteria located in the interior regions of the biofilm (27). These dead bacteria then served as a nutrient source for the bacteria located in the exterior regions of the biofilm, allowing them to survive and proliferate. This dynamic supports the maintenance and expansion of the biofilm even under nutrient-limited conditions, highlighting the complexity of bacterial survival mechanisms within biofilms.

These discussions emphasize the intricate dynamics of bacterial communities and their interactions with bacteriophages, highlighting how microbial ecosystems, whether in the gut, oral cavity, or biofilms, influence infection dynamics. Additionally, the findings offer insights into how bacteria resist phage predation, which could inform future therapeutic strategies targeting biofilms and phage resistance.

## FUNGI AND DRUG RESISTANCE

Infections caused by environmental fungal pathogens have increased sharply over the last two decades, resulting in over 3.5 million deaths annually, with average mortality rates exceeding 20%. Dr. Linqi Wang from the Institute of Microbiology, Chinese Academy of Sciences, introduced his work on the environmental adaptation of human fungal pathogens. His research explores why environmental fungi like *Cryptococcus neoformans* cause fatal infections and exhibit treatment resistance. Key findings include glucose-driven phenotypic resistance mediated by transcription factor Mig1, compromising amphotericin B efficacy, while blocking IPC synthesis enhances therapeutic outcomes (15). In lung infections, a glucose-poor condition, *Cryptococcus* may form dormant cell populations to tolerate Amphotericin B treatment. The antidepressant sertraline was identified as a promising adjunct therapy. Additionally, global warming accelerates fungal mutagenesis, driving the emergence of new pathogenic strains developing pan-drug resistance to fluconazole, caspofungin, and amphotericin B, but not polymyxin B (28). These insights provide innovative strategies for combating fungal infections and highlight the impact of environmental factors on fungal evolution and drug resistance.

Fungi have co-evolved with humans for thousands of years, colonizing various body sites such as the oral cavity, gastrointestinal tract, and genital regions. Dr. Bing Zhai from Shenzhen Institute of Advanced Technology, Chinese Academy of Sciences, explores how endogenous fungi, particularly *Candida* species, interact with the human body, focusing on immunocompromised patients and pregnant women (29). *Candida parapsilosis* was found to colonize the gut before causing bloodstream infections, with hetero-resistance (30). A subpopulation of *Candida* survives high drug concentrations, emerging as a key driver of breakthrough infections globally. Additionally, *Candida glabrata* demonstrated *in vivo* evolution under drug pressure, acquiring resistance through point mutations. Dr. Zhai’s research highlights the critical need to monitor fungal colonization, understand resistance mechanisms in real-world settings, and develop innovative strategies to combat fungal infections.

To address the growing threat of fungal pathogens, Dr. Tao Dong from Southern University of Science and Technology, China, presented their recent findings demonstrating how bacterial systems can effectively combat these pathogens. His research focuses on the Type VI Secretion System (T6SS), a bacterial protein delivery mechanism capable of killing fungal pathogens, including the drug-resistant *Candida auris*, a significant clinical concern. T6SS operates by injecting effector proteins, such as TseN, which is identified by mutant screening, into fungal cells. TseN enters the fungal nucleus via a nuclear localization signal, where it disrupts DNA via its C-terminal domain and induces cell death. In addition, he found that *Candida* species can partially resist T6SS by forming protective hyphae. To overcome this resistance, combining T6SS with drugs that inhibit hyphal formation could enhance its efficacy. Furthermore, his study demonstrates the potential of genetically engineered *E. coli* expressing functional T6SS as a versatile antimicrobial tool. Ongoing efforts aim to optimize T6SS efficiency for broader pathogen control, offering promising advancements for human and environmental health treatments.

## VIRAL STRUCTURE AND PATHOGENESIS

Dr. Zihe Rao from Tsinghua University, China, opened the discussion with his extensive research on elucidating the structures of crucial viral components across multiple pathogens, including influenza, coronaviruses, flaviviruses, and hepatitis A and B. Dr. Rao then focused on a recent breakthrough study that explored the structural dynamics of the hepatitis B virus surface antigen (HBsAg), a critical protein responsible for HBV invasion, replication, and packaging within host cells. Dr. Rao and his team invested 20 years of effort, utilizing an advanced single-particle cryo-electron microscopy and extensive data sets, to ultimately decipher the near-atomic resolution (3.7 Å) 3D structure of HBsAg (31). They successfully mapped the structural flexibility of the hepatitis B surface protein and its self-assembled forms, uncovering the molecular basis of the diverse oligomeric formations on the virus particle surface. Through detailed analysis of one typical trimer and three distinct tetramer assembly patterns on subviral particles, this team elucidated how spherical particles extend into tubular filaments and interact with the nucleocapsid to form complete virions. This work addresses long-standing questions in structural biology and virology but also provides critical insights for vaccine design and neutralizing antibody discovery. Notably, it holds the potential to accelerate the development of small molecules targeting the viral surface protein and envelope assembly, including PROTAC (PROteolysis Targeting Chimera) compounds that guide surface protein degradation.

In addition to the viral surface protein, viral inclusion bodies (IBs) serve as critical sites for viral replication and assembly, yet their formation mechanisms remain unclear. Using a combination of biophysical and cellular approaches, Dr. Chao Wu from Shenzhen Bay Laboratory, China, reveals that the Ebola nucleoprotein (eNP) and viral protein 35 (eVP35) form the eNP–eVP35 complex, which phase separates to drive IB formation (32). The process is reversible, modulated by ionic strength, and critically dependent on the multivalency of eVP35 rather than eNP. eNP plays a pivotal role in nucleocapsid remodeling, a rate-limiting step in viral replication and transcription, by binding to single-stranded RNA (ssRNA), forming ring-like structures, and packaging ssRNA for replication. Overexpression of an eVP35 peptide disrupts the eNP–eVP35 complex formation, reducing IB formation and limiting viral infection, suggesting a potential therapeutic strategy targeting nucleocapsid remodeling.

## HOST–VIRUS DYNAMICS

Viruses are highly adaptive pathogens that rely on intricate interactions with host cells to replicate, evade immune detection, and ensure their survival. The interactions between the host and virus determine the outcome of infection and influence viral evolution, transmission, and the emergence of drug resistance. By exploring how viruses manipulate host cellular machinery, immune responses, and metabolic pathways, researchers can uncover novel targets for antiviral therapies, vaccines, and immune interventions. Several presentations in this meeting showcase recent breakthroughs in host–virus dynamics, offering valuable insights into the molecular mechanisms underlying viral infection and persistence and highlighting the potential for innovative therapeutic approaches.

Influenza virus is one of the highly contagious pathogens due to its high mutation rate and ability to infect multiple species. Primarily caused by influenza A and B types, the virus triggers seasonal outbreaks and can lead to severe illness or death, especially in high-risk groups. Dr. Stacey Schultz-Cherry from St. Jude Children’s Research Hospital, USA, presented findings on the impact of obesity on influenza virus infection, showing that obese hosts exhibit more severe symptoms and prolonged viral shedding. Mechanistically, obesity is associated with chronic low-grade inflammation and an upregulation of ROS, both of which contribute to a compromised antiviral defense. Obese hosts exhibit a blunted interferon response due to increased degradation of TLRs via upregulated integrin proteins, leading to reduced interferon signaling and lower expression of interferon-stimulated genes. This dampened immune response allows the virus to replicate more efficiently and establish infection in both upper and lower respiratory tracts, where obese animals show higher viral loads and extended virus shedding compared to lean animals. Additionally, increased viral replication in an obese host facilitates the generation of diverse viral populations, raising the risk of mutations that can lead to drug resistance or immune escape, ultimately enhancing the virus’s ability to evolve and transmit more effectively. The findings emphasize that metabolic health, rather than obesity alone, is crucial in influencing viral evolution and guiding effective influenza control measures. Dr. Yuelong Shu from Chinese Academy of Medical Sciences and Peking Union Medical College investigates the host factors that influence human susceptibility to influenza viruses, particularly H7N9, which has caused severe outbreaks with high fatality rates. Genetic analysis of avian IAV H7N9 patients highlighted the MX1 gene, an interferon-induced antiviral guanosine triphosphatase, showing that a rare, heterozygous single-nucleotide mutation in this gene exerted a dominant negative effect on the antiviral function (33). Additionally, epidemiological data revealed that H7N9-infected men had significantly reduced testosterone levels, which were linked to worse outcomes and higher levels of proinflammatory cytokines like IL-6 and IL-15. Preliminary findings also suggest that the gut microbiome influences susceptibility, with some bacteria offering protection and others increasing risk. These complex host factors and their interactions with environmental elements underscore the need for better models to fully understand the mechanisms of avian influenza infection and inform public health interventions.

Flaviviruses, including dengue, Zika, yellow fever, and West Nile viruses, are RNA viruses that pose global health threats due to their ability to cause severe diseases and rapid geographic spread, primarily through mosquito or tick vectors. The lack of effective antiviral treatments and vaccines for many flaviviruses further compounds their danger, requiring urgent advancements in prevention and control strategies. Dr. Gong Cheng from Tsinghua University, China, presented recent findings on vector-borne flaviviruses, exploring the role of gut bacteria in regulating flavivirus transmission in mosquitoes. By isolating various bacterial strains from field-collected *Aedes albopictus* mosquitoes, they identified a key bacterium, *Rosenbergiella* sp. YN46, which plays a crucial role in modulating the mosquito gut environment, thereby impacting viral entry into cells (34). This bacterium was shown to secrete glucose dehydrogenase (RyGDH), lowering the gut pH, which disrupts flavivirus envelope protein and reduces mosquito susceptibility to infection. Experimental results demonstrated that introducing *Rosenbergiella* sp. YN46 into mosquito breeding water led to its colonization in the mosquito gut, significantly reducing viral loads in mosquitoes. This suggests that incorporating *Rosenbergiella* sp. YN46 into mosquito habitats could serve as a biocontrol strategy to limit flavivirus transmission, providing a promising approach for managing vector-borne diseases like dengue in endemic regions. Dr. Qiang Ding from Tsinghua University, China, uncovered the mechanisms by which the ZIKA virus evades the host immune response, specifically through viral NS5-mediated STAT2 degradation, thus impairing type I interferon (IFN)-mediated antiviral immunity. His team utilized a genome-wide CRISPR/Cas9 screen, which identified ZSWIM8 as the substrate receptor of the Cullin3–RING E3 ligase complex essential for NS5-mediated STAT2 degradation (35). Depletion of ZSWIM8 in cells led to partial resistance against ZIKV infection, preserving STAT2 levels and enhancing IFN signaling, thereby reducing viral infection. His research provides novel insights into the interaction between ZIKV and host factors, highlighting the role of NS5 in reprogramming the ZSWIM8–CUL3 E3 ligase complex to degrade STAT2, and it holds potential for developing antiviral strategies and vaccines.

In contrast to acute infection, some viruses, instead of being cleared completely by the immune system, establish latency by integrating into the host’s genome or persisting as episomes in specific cell types. In this latent state, viral activity is minimized, allowing the virus to avoid detection while retaining the potential for reactivation, ensuring long-term survival within the host. Two speakers discuss the viral latency established by herpesviruses in the meeting. Dr. Felicia Goodrum from University of Arizona, USA, shared her study on cytomegalovirus (CMV), a persistent herpesvirus that establishes lifelong infections and poses significant health risks in immunocompromised individuals. Her research focuses on understanding the mechanisms of CMV latency and reactivation, with an emphasis on specific viral genes in the ULb' region, including UL138, UL135, and UL136, using a humanized mouse model generated by injecting CMV-infected human fibroblasts into immunocompromised NOD mice. Notably, the UL136 gene encodes multiple protein isoforms, each with distinct roles in latency. One isoform, P33, is highly unstable. Its degradation is essential for CMV latency but not viral replication (36). Dr. Goodrum found the host factor IDOL targets P33 for degradation, while overexpression of IDOL led to loss of P33 but not lysine-mutated P33 (37). Further investigations in THP-1 monocyte cell models showed that IDOL is highly expressed in undifferentiated cells but downregulated during differentiation, pointing to the necessity of P33 degradation for latency maintenance. Additionally, the drug GW3965, which activates LXR and IDOL, promotes P33 instability, thus restricting viral reactivation from latency. These findings reveal how CMV exploits host pathways to regulate latency and reactivation, providing promising insights for therapeutic strategies in controlling persistent viral infections. Dr. Ke Lan from Wuhan University, China, presented his findings on how Kaposi’s sarcoma-associated herpesvirus (KSHV) regulates latency and lytic replication within host cells, highlighting the crucial role of the host protein NDRG1 in the virus’s lifecycle. After initial infection, KSHV typically enters a latent state, expressing only a limited set of genes. However, under conditions such as immunosuppression, hypoxia, or stress, the virus can be reactivated and enter lytic replication. The RTA protein acts as the main regulatory factor to initiate lytic replication, while the LANA protein maintains latency by anchoring the viral genome to the host chromosomes and suppressing RTA’s auto-activation (38). The study reveals that NDRG1 is significantly upregulated in KSHV-infected cells and forms a complex with LANA and PCNA (proliferating cell nuclear antigen), which helps load PCNA onto the viral genome to facilitate its replication (39). Additionally, during lytic replication, NDRG1 interacts with ORF44, enhancing its stability and thereby supporting viral genome replication and maintaining multiple genome copies (40). This mechanism indicates that NDRG1 plays a critical role in both the latent and lytic stages of KSHV replication, offering new insights into viral replication control and potential antiviral therapeutic strategies.

Dr. Ying Lin from Chinese Academy of Medical Sciences & Peking Union Medical College presented her research on the role of host factors in human adenovirus B7 (HAdV-B7) infections. Through a high-throughput gain-of-function screen of over 15,000 host factors, Dr. Lin identified glycogen synthase kinase 3 α (GSK3A) as a key proviral factor that enhances HAdV-B7 replication. Her results showed that GSK3A expression significantly increased following HAdV-B7 infection; overexpression of GSK3A promoted viral replication, while its knockdown or knockout inhibited replication. Importantly, the kinase activity of GSK3A was essential as it facilitates phosphorylation of the viral protein L4-22K. Mutations that disrupted GSK3A’s interaction with L4-22K altered replication patterns, highlighting this pathway as a potential therapeutic target.

## VIRAL VACCINE AND ANTIVIRAL THERAPEUTICS

Viruses pose a significant threat to global health due to their ability to cause widespread outbreaks, high mutation rates, and rapid transmission. They can lead to severe illness, overwhelming healthcare systems, and economic disruptions. Emerging and re-emerging viruses, such as SARS-CoV-2 and influenza, highlight the urgent need for global preparedness and effective prevention, treatment, and control strategies. Several speakers presented advancements in vaccine design and antiviral drug development, emphasizing the need for broad-spectrum immunity and cross-variant protection.

SARS-CoV-2 vaccine development encounters significant challenges, including the attenuation of immune responses over time and diminished efficacy against rapidly evolving variants. Dr. Linqi Zhang from Tsinghua University, China, shared research studies on the immune response of a unique cohort infected with SARS-CoV in 2003 and later with SARS-CoV-2 in 2023, aiming to uncover insights into long-term immune memory and cross-protective immunity (41). This study, spanning two decades, focuses on how immunity adapts to sequential infections and the implications for antibody and vaccine development. Using five distinct cohorts that included both primary and breakthrough infections, Dr. Zhang and his team found that prior infection or vaccination significantly broadened antibody responses, enhancing both neutralization potency and breadth across SARS-CoV-2 variants. Antigenic mapping and monoclonal antibody isolation techniques revealed that breakthrough infections are particularly effective in expanding antibody breadth against diverse SARS-CoV-2 strains. These findings indicate that immune memory from previous infections may enhance cross-variant protection and suggest that regular exposure could strengthen immunity without the need for variant-specific vaccine updates. Dr. Zhang concluded that these insights could be pivotal for next-generation vaccine strategies, highlighting the value of immune adaptability and memory in ongoing SARS-CoV-2 protection efforts. Dr. Zheng Zhang from Shen Zhen Third People’s Hospital, China, further explored immune responses across SARS-CoV-2 infections, focusing on identifying long-lasting T- and B-cell responses to develop broad-spectrum immunity. Through longitudinal SARS-CoV-2 studies, he found that while antibody levels generally decline over time, certain antibodies maintain high levels even 2 years post-infection. These long-lasting antibodies frequently utilize V_H_ segments like V_H_3-53 and V_H_3-66, which effectively block ACE2 binding and show broad neutralization. Additionally, they found T-cell responses were shown to play a critical role in combating viral infections. In hepatitis B virus (HBV) studies, they found that specific T-cell subpopulations were associated with higher functional cure rates, particularly in younger patients with low viral loads. His work provides insights into designing vaccines and therapeutic strategies that leverage long-term and cross-variant immunity.

Dr. Ren Sun from Westlake University, China, shared advancements in the rational design of vaccines using a high-resolution functional map to enhance viral immunogenicity. By systematically identifying and targeting immune evasion functions within viral genomes, Dr. Sun has developed viruses with increased sensitivity to immune responses, notably interferon and cell death pathways (42). In engineered influenza virus strains, HIS (hyper-interferon-sensitive) mutations prevent viral replication in the host while generating strong immune responses, serving as an effective preventive vaccine model. Additionally, Dr. Sun explored the therapeutic application of these engineered viruses in cancer immunotherapy by injecting HIS into tumors to stimulate local immune responses, synergizing effectively with immune checkpoint inhibitors like anti-PD1. Dr. Sun also introduced their newly developed high-throughput method to measure antibody responses against thousands of viral antigens, helping pinpoint specific immune responses to viral mutations and the immunogenicity of antigens. This comprehensive approach not only optimizes vaccine efficacy but also opens new avenues for oncolytic virotherapy and the development of broadly protective vaccines.

Developing an effective dengue vaccine require broad immunogenic coverage to prevent severe secondary infections caused by antibody-dependent enhancement. Dr. Ana Fernández-Sesma from Icahn School of Medicine at Mount Sinai, USA, outlined current vaccines, including Takeda’s and Butantan’s vaccines, highlighting their limitations and efficacy. Her works focus on understanding immune responses across serotypes, using monocyte-derived dendritic cell (MDDC) models to assess attenuation and immunogenicity. The DENV4 tetravalent vaccine induces high levels of pro-inflammatory cytokines and achieved 100% protection. Phase III trials also report 80% protection with tetravalent formulations, although durable immunity requires T-cell assistance to support long-lived, germinal center (GC)-experienced B cells. This research underscores the need for T-cell assistance and long-lasting antibody responses against dengue’s structural and nonstructural proteins to optimize vaccine design.

Dr. Deyin Guo discussed his research efforts focused on developing antiviral drugs, particularly for respiratory viruses, through innovative strategies at Guangzhou National Laboratories in China. Emphasizing the need for broad-spectrum antiviral drugs that can target entire virus families rather than individual strains, Dr. Guo highlights the challenges posed by emerging viruses and the limited number of effective antivirals currently available. By studying critical viral mechanisms like RNA replication and methylation, the team has identified promising drug targets and developed candidate molecules, such as a modified inhibitor of RNA polymerase and methyltransferase, which have shown efficacy in preclinical and clinical trials. This approach aims to enhance preparedness against future pandemics by creating adaptable treatments for viral outbreaks.

Dr. Min-hua Luo from Wuhan Institute of Virology, Chinese Academy of Sciences, explored the mechanisms of virus-induced neural damage and intervention strategies, including tracing techniques for trans-synaptic neuron circuits to analyze connectivity and function. Emphasis is placed on developing large-capacity viral vectors for gene therapy and creating bright, visible markers for viral infection tracking. Using HSV-1 strain H129 viral tracing models, Dr. Luo has been able to visualize how viruses spread through neural circuits and interact at synaptic levels, addressing key questions about viral axonal transport and presynaptic secretion (43). Additionally, using the CMV viral tool, Dr. Luo focuses on congenital infection models to study brain development and trace brain infection and damage (44). By improving visibility and monitoring through MR and other imaging systems, the aim is to track infection progression and brain damage, providing a basis for better diagnostics and early treatment strategies.

## EPIDEMIOLOGY AND SURVEILLANCE OF VIRUSES

The closing presentations addressed the evolutionary dynamics of viruses and the importance of continued vigilance. Dr. Zhengli Shi from Guangzhou Laboratory, China, explored the diversity and evolution of SARS-related coronaviruses, emphasizing the need for ongoing surveillance. Her work reveals that SARS-CoV and SARS-CoV-2 share structural similarities in their genomes, but SARS-CoV-2 has a unique protease cleavage site on its spike protein, enhancing its transmissibility and leading to the global pandemic. Evolutionary analysis and recombination events identified in coronaviruses found in bats show high variability in the nonstructural protein and spike protein regions (45). Experiments also demonstrate that viruses with specific mutations require high concentrations of trypsin to infect cells, potentially related to their binding capacity with the human ACE2 receptor. Additionally, coronaviruses from different sources exhibit significant differences in neutralizing activity, suggesting that future vaccine development must account for genomic and antigenic diversity among strains. This research emphasizes the importance of ongoing surveillance of coronaviruses in nature and in environments where humans and domesticated animals coexist, to facilitate early detection and prevention of potential zoonotic infections.

Dr. Wenhong Zhang from Huashan Hospital, Fudan University, China, discussed the evolution and control of infectious diseases in human history, including acute infections (like polio and smallpox) and persistent infections (like HBV and HIV). While traditional vaccines effectively eliminated some acute infections, managing persistent infections remains a challenge, especially with the emergence of new pathogens like COVID-19. The discussion highlighted advances in vaccine technologies, including mRNA vaccines and heterologous vaccination strategies, which have shown promise in expanding immunity. Future pandemic-preparedness should prioritize rapid vaccine development, aiming for broad-spectrum vaccines and a 100-day readiness to combat novel pathogens. Additionally, new therapeutic strategies combining direct-acting agents with immune enhancement are essential to manage chronic infections such as HBV. This strategy may pave the way toward preventing and potentially eradicating both emerging and existing infectious diseases.

## CONCLUSION

This meeting highlighted significant advancements in bacteriology, fungi, virology, immune response mechanisms, and therapeutic interventions. The diverse research shared provides new insights into antimicrobial resistance, pathogen–host interactions, immune modulation, bacterial community, viral pathogenesis, and the ongoing quest to design effective vaccines and antiviral therapies. The discussions underscored the importance of next-generation antibiotics, host-directed therapy, vaccines with increased efficacy, and the need for innovative approaches in both pandemic preparedness and chronic infection management. The collective work presented here lays the groundwork for future research and translational applications that could transform the management and prevention of infectious diseases globally.

## AFTER EPUB

*[This article was published on 24 February 2025 with an error in the co-chair's name. The name was corrected in the current version, posted on 3 March 2025.]*
